# Polymerase chain reaction is superior to serology for the diagnosis of acute Mycoplasma pneumoniae infection and reveals a high rate of persistent infection

**DOI:** 10.1186/1471-2180-8-93

**Published:** 2008-06-11

**Authors:** Anna C Nilsson, Per Björkman, Kenneth Persson

**Affiliations:** 1 Department of Clinical Sciences, Malmö, Infectious Disease Research Unit, Lund University, Malmö University Hospital, Sweden; 2 Clinical Microbiology, Lund University, Malmö University Hospital, Malmö, Sweden

## Abstract

**Background:**

Diagnosis of *Mycoplasma pneumoniae *(MP) infection is traditionally based on serology, which may require more than two weeks for diagnostic antibodies to develop. PCR-based methods offer earlier diagnosis. During a community outbreak of MP infection, we compared semi-nested and real-time PCR of oropharyngeal swabs with serology for diagnosis of MP infection at different time points after disease onset. PCR-positive individuals were followed longitudinally to assess the persistence of MP DNA in throat secretions. We also studied carriage of MP among household contacts and school children.

**Results:**

MP infection was diagnosed in 48 of 164 patients with respiratory tract infection. Forty-five (29%) had detectable MP DNA in oropharynx. A significant increase in MP IgG IgG titre or MP IgM antibodies was detected in 44/154 (27%) subjects. Two MP PCR-positive patients lacked antibody responses. Sera were missing from another two patients. The agreement between serology and PCR was good, κ = 0.90.

During the first three weeks after disease onset the performance of PCR was excellent and all patients but one were detected. In contrast, only 21% of the patients with confirmed MP infection were positive by serum 1 during the first symptomatic week (56% during the second and 100% during the third week). Only 1/237 (0.4%) school children was positive by PCR. This child had respiratory symptoms. Eighteen of 22 (75%) symptomatic household contacts were MP PCR positive.

Persistence of MP DNA in the throat was common. Median time for carriage of MP DNA was 7 weeks after disease onset (range 2 days – 7 months). Adequate antibiotic treatment did not shorten the period of persistence. Bacterial load, measured by quantitative real-time PCR declined gradually, and all followed patients eventually became PCR-negative.

**Conclusion:**

PCR is superior to serology for diagnosis of MP infection during the early phases of infection. Persistent, sometimes long-term, carriage of MP DNA in the throat is common following acute infection, and is not affected by antibiotic therapy. Asymptomatic carriage of MP even during an outbreak is uncommon.

## Background

*Mycoplasma pneumoniae *(MP) is a small bacterium without a cell wall. It is recognized as a common cause of community-acquired pneumonia and upper respiratory tract infections, especially in children and adolescents, although all age groups may be affected. MP infections tend to occur in epidemics with a predilection for clustering in families and groups with close contacts such as military conscripts [1,2]. Following an incubation period of two to three weeks, the infection is characterised by respiratory symptoms with cough, fever and malaise. MP infection is usually self-limited, but treatment with antibiotics such as erythromycin, tetracycline or quinolones is often prescribed.

Traditionally, diagnosis of MP infection has been based on serology, using either a rise of IgG titre in paired sera, or the detection of MP IgM in acute phase serum. However, antibodies may not appear until two weeks after the onset of symptoms, and may thus provide a diagnosis only retrospectively in many cases [3]. Apart from low sensitivity in acute disease, serological tests may also have specificity problems [4]. Direct methods for diagnosing MP infection have therefore been considered. Culture of MP is difficult to perform, takes a long time and is not suitable for clinical practice. Instead, detection by PCR from respiratory secretions has been suggested as a more sensitive and practical diagnostic tool [5-8]. PCR methods targeting the adherence protein P1 or the 16S RNA gene, as well as other genes have been described [7,9-15]. Most comparative studies of serology and PCR have included small numbers of MP-positive cases [7,10,16-18] and only rarely has it been possible to evaluate the performance of the tests at different intervals after onset of illness [4]. Furthermore, asymptomatic carriage could complicate the assessment of a positive PCR finding. Rates of MP carriage in healthy people have been reported to be between 0–13.5% [7,8,19,20].

MP infection can sometimes cause persistent respiratory symptoms, as well as a range of late-stage extra-pulmonary complications. The aetiology of these manifestations is not clear; although immune-mediated pathogenesis may be responsible, long-term MP infection could also be involved.

This study compares the performance of DNA detection by PCR and serology at different time points after onset of symptoms in MP infection. To assess the prevalence of asymptomatic carriage, school children were examined during a community outbreak of MP infection. A longitudinal follow-up was also performed in PCR-positive patients to determine the rate of bacterial clearance. In addition, the prevalence of carriage of MP was studied among household contacts to some of the patients.

## Methods

### Material

The study was performed in the city of Malmö and adjacent suburban areas (pop. approx. 360 000) in the south of Sweden between September 20, 2005 and March 15, 2006. During this period an increased number of MP infections were noted in the diagnostic serological laboratory at the Department of Clinical Microbiology. Three groups of subjects were included in the study: patients with symptoms compatible with MP infection, household contacts of MP-infected patients, and school children healthy enough to be in school.

Patients with acute respiratory symptoms including cough were eligible for inclusion. No exclusion criteria were applied. Study participants were recruited from four primary health care centres in Malmö and from the Department of Infectious Diseases at Malmö University Hospital. One hundred-sixty-four patients were included (62% women, 38% men; median age 41 years [range 2 – 82]).

Children aged 10–16 years; were recruited from a primary school in Malmö. Two hundred thirty-seven children were included, and had a throat swab for PCR taken on one single day. Additionally 22 out of 25 household contacts to ten positive index cases in ten different families were investigated by PCR.

### Study procedure

Samples for PCR testing were obtained from oropharynx at the posterior pharyngeal wall by a cotton-tipped swab and transferred to a tube containing either a transport medium consisting of 1 mL of phosphate buffered saline supplemented with bensylpenicillin 150 μg/mL, gentamicin 10 μg/mL and amphotericin 2 μg/mL, or a commercial transport medium (Copan Italia SPA, Brescia, Italy).

Serum samples were collected on the first visit and at least 2–4 weeks later.

Patients and household contacts were asked to provide both throat samples and a blood sample for serology, whereas only throat samples were obtained from school children. Household contacts and school children answered questionnaires concerning disease symptoms at the time of sampling and during the preceding weeks.

Subjects who were positive by PCR were invited to follow-up PCR testing every two weeks until two consecutive samples were negative. Three to six months after having submitted the last PCR-negative throat sample, these individuals were asked to return for another throat PCR sample in order to evaluate potential bacterial recurrence.

### Methods

#### PCR

Samples for PCR analysis were placed in tubes containing phosphate buffered saline (PBS), and DNA was extracted using a MagNa Pure LC instrument and a MagNa Pure LC DNA I isolation kit (Roche Diagnostics GmbH, Mannheim, Germany). Nucleic acid from a 200-μL aliquot of each sample was finally obtained in 75 μL elution buffer, and a semi-nested PCR was performed using primers described elsewhere [21]. Amplified DNA was subjected to gel electrophoresis after a characteristic amplification product detected by ethidium bromide staining. The following primers were used:

MP11 5'-TGC CAT CAA CCC GCG CTT AAC

MP12 5'-CCT TTG CAA CTG CTC ATA GTA

MP-I 5'-CAA ACC GGG CAG ATC ACC TTT

Semi-nested PCR was carried out using MP11/MP12 followed by MP12/MP-I, which yields a 201-bp-long fragment. A PCR mixture (total volume 50 μL) was prepared that included 10 μL of the sample containing each nucleotide at a concentration of 200 nM, each primer at 500 nM, 2 μM MgCl_2_, and 1.5 u Taq-polymerase (Go-Taq, Promega, Madison, WI, USA).

A quantitative real-time PCR (qPCR) method was used to measure the load of MP at different time points in patients with positive reactions in semi-nested PCR. Primers were MP 435 5'-GGCAGTCAACAAACCACGTATG and MP 479 5'-GGTGGTTGATGCGGTCAAA with the probe MP 509 FAM-5'-CCCACCCGAACCGAAGCGG-TAMRA. The amplification fragment was cloned into a plasmid vector, which was used as a standard for quantification. Real time PCR was performed using a GeneAmp 5700 Sequence Detector (Applied Biosystems, Foster City, CA, USA). A PCR mixture (25 μL) was prepared that included 5 μL of the sample containing each primer at 400 nM, 250 nM probe and each nucleotide at 200 nM, and 1.5 u GoldTaq (Applied Biosystems/Roche, Branchburg, NJ, USA)

#### Serology

IgG antibodies were measured using the Serion Elisa Mycoplasma pneumoniae IgG kit (Institut Virion/serion GmbH, Würzburg, Germany) with the test cut-off 0,5×mean optical density (OD) value of the kit control serum, as indicated in the insert. A positive IgG reaction was defined as >30 AU/mL. A significant rise in IgG titre was considered to be a doubling of the OD value above the cut-off, or a sero-conversion in which the primary serum was antibody negative and the second serum had an OD at least twice the cut-off corresponding to a threefold rise in AU/mL titre. IgM antibodies were measured using the SeroMP™ IgM kit (Savyon^® ^Diagnostics Ltd. Ashdod, Israel). A calibration line obtained by the standard sera in the kit was used to determine the IgM titre in BU/mL. A positive IgM antibody reaction was defined as >20 BU/mL according to the instruction of the manufacturer.

### Gold standard and discrepant analysis

Diagnosis of MP infection was based on serology or PCR findings. A significant rise in MP IgG or seroconversion in paired sera or the presence of IgM antibodies to MP were used as sufficient criteria of current MP infection. MP infection was also considered to be present by DNA detection when two independent PCR methods were positive. In cases with positive PCR results in the absence of MP antibodies, sequencing was done to confirm the presence of MP infection. Two consecutive samples negative by PCR were considered a microbiological resolution of MP infection.

### Statistical analysis

Agreement of the results was calculated using the kappa index. Differences between mean values and between frequencies were tested by the Mann-Whitney U test and the chi-square test, respectively. Persistence of *M. pneumoniae *DNA was studied by survival analysis according to Kaplan-Meier. The Statistica package was used (StatSoft Inc., Tulsa OK, USA).

### Ethical considerations

The ethics committee of the University of Lund approved the study, and written informed consent was obtained from all patients or from their caretakers.

## Results

### Comparison of PCR and serology

Among 164 patients included in the study, 45 (27%) were positive by PCR in throat swabs on at least one occasion (Table 1). Sera were available for 154 patients, of whom 44 (29%) had a significant antibody response. For 96 patients with paired sera and throat swabs, the agreement between serology and PCR was κ = 0.90. Three PCR-positive cases (11, 33 and 79 years old) had unexpectedly late serological responses. Their first serum samples were obtained during the fourth week of illness when they had low IgG titres and undetectable IgM. MP infection was serologically confirmed by IgG titre rise in convalescent phase samples obtained on days 41, 58, and 80, respectively.

**Table 1:** MP infection diagnosed by PCR and serology in 164 patients with respiratory tract infection.

Pcr reaction	Patients with paired sera		Patients with single sera		Total serum results		Patients without sera	Total
					
	Ser+*	Ser-**	Ser+***	Ser-**	Ser+*	Ser-**		
PCR+	25	2	16	0	41	2	2	45
PCR-	2	67	1	41	3	108	8	119
Total	27	69	17	41	44	110	10	164

* = IgM+ or IgG seroconversion

** = IgM- and IgG-

*** = IgM+

The time interval between onset of illness and first sample was essential for the sensitivity of serology. During the early stages of illness PCR was clearly superior to serology in identifying MP. Only 21% of the MP cases had a diagnostic antibody reaction in their acute phase serum obtained during the first week of illness, whereas all tested patients with confirmed MP infection were positive at that time. During the second week of illness, 56% of the MP cases were detected by serology and 96% by PCR (Table 2).

**Table 2:** Performance of serology and PCR at first visit in patients with MP infection in relation to duration of illness.

Days after onset of illness	MP cases detected by PCR (%)		MP cases detected by serology. Results of the first serum (%)	
		
	Negative	Positive	Negative	Positive
1 – 7	0	17 (100)	10	3 (23)
8 – 14	1	23 (96)	8	10 (56)
15 – 21	0	8 (100)	0	6 (100)
22 – 28	2	5 (71)	5	1 (17)
> 28	0	8 (100)	2	9 (82)
Total	3	61 (95)	25	29 (54)

Patients who presented during the third week of illness were all positive by PCR and serology. The sensitivity of PCR declined in the small number of patients who had their first test later than three weeks after onset of symptoms.

Quantitative PCR could be carried out in 44 of the 45 patients who were positive by semi-nested PCR. One sample contained insufficient material for analysis but the infection was confirmed by IgG titer rise. qPCR confirmed MP infection in 42/44 cases while two cases tested negative by this method. These two cases were further analysed by DNA sequencing and found to belong to genotype 2 of MP thus confirming MP infection.

### Discrepant findings of PCR and serology

Two patients had repeated samples positive by PCR but did not develop antibody responses. Both had symptoms suggestive of MP infection. One of them was pregnant in the third trimester. Serum samples were collected from this patient (age 29 years) on days 30, 46 and 60 after onset of symptoms (PCR positive on days 30 and 46) and from the other patient (age 53 years) on days 1 and 36 (PCR positive on days 1 and 7). The nested PCR results were confirmed by qPCR and also by sequencing the MP genome. Three other patients (79, 34 and 69 years old) had serological responses indicating MP infection but were negative by PCR. Sera and samples for PCR were taken on days 25/58, 27/41 and 11 respectively. The first two patients had a significant titre rise of IgG antibodies. The third patient had a positive IgM reaction for MP on day 11 but lacked IgG antibodies at that time.

### Bacterial load by quantitative PCR (qPCR)

The qPCR was compared to the established semi-nested PCR in 142 clinical samples obtained for routine testing of MP infection. The semi-nested PCR detected 24 positive cases (17%) and the qPCR 23 of the 24 positive cases. The sensitivity and specificity for the qPCR were 96% and 100% respectively.

**Figure 1:**
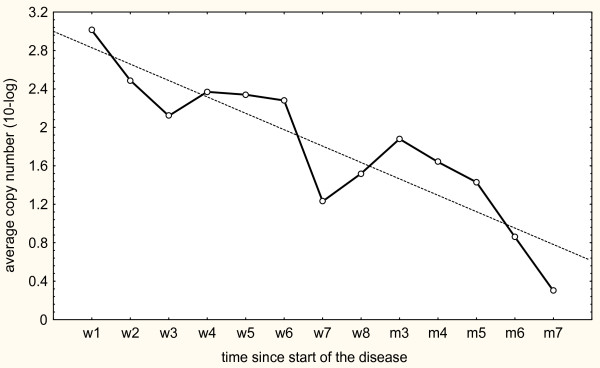
**Geometric mean values of MP load at different time points after onset of illness.** The results are based on the longitudinal follow-up of 53 patients.

The patients of the present study were also followed longitudinally by qPCR. The bacterial load in consecutive samples gradually declined in relation to the time interval from onset of illness to sampling. Figure 1 shows the geometric mean copy numbers of organism DNA observed during each week and a calculated regression line.

Forty-three of the individuals followed longitudinally were prescribed antibiotic treatment active against MP on basis of clinical symptoms by the attending physician. The treatment of choice was erythromycin, roxitromycin, tetracycline or levofloxacin. Ten patients did not receive any antibiotic treatment. The median period of persistence of MP among the 43 cases treated with antibiotics was 52 days (range 2–229) compared to 45 days (range 1–116) among the 10 untreated cases. No difference in persistence was demonstrated between these groups using the Mann-Whitney U test, p = 0.16.

### Long term persistent infection

**Figure 2:**
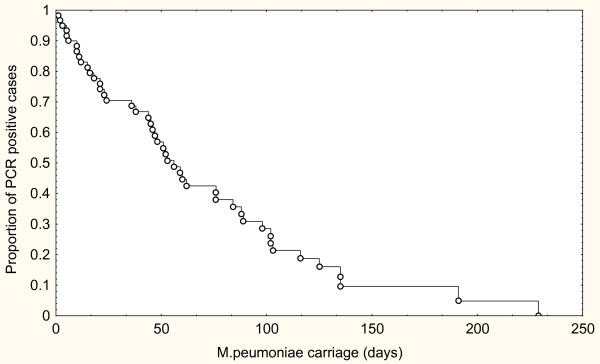
Decline of carriage rate of *M. pneumonaiae *over time reflected in a Kaplan-Meier curve based on 60 PCR positive patients.

Results from follow-up samples by PCR and qPCR were complete in 46 individuals (patients and family contacts) and incomplete in an additional 14 cases. A survival curve according to Kaplan-Meier for these 60 PCR positive cases is shown in Fig. 2. Long-term persistence of MP was common after the acute phase of the illness. Half of the cases were positive after 54 days.

Median follow-up time of the individuals was 138 days, range 11–337 days. Late samples were obtained from 33 patients three to six months after microbiological resolution of their infections, and 32 of those were PCR negative. One case had become positive again after two negative samples three months earlier, but eventually became negative after an additional three months.

### School children

Among 237 school children, 236 were negative by MP PCR in throat swabs. One 14-year-old boy was PCR-positive, and subsequently tested positive in repeated samples obtained over the following three months. This boy was found to have symptoms of respiratory tract infection compatible with MP infection, and his condition improved by treatment with erythromycin. All school children were also tested for *Chlamydophila pneumoniae *by PCR but no such infection was found.

### Family contacts

In 18 of the 22 tested family members in ten families with an index case MP DNA was detected by semi-nested PCR and quantitative PCR. All PCR-positive family members had ongoing or recent respiratory tract symptoms at the time of testing. One child was healthy and negative on initial testing, but later developed symptoms and became PCR positive.

## Discussion

Although rarely fatal, *Mycoplasma pneumoniae *is an important cause of acute respiratory tract infection, especially as a potential aetiology of the clinical entity termed "atypical pneumonia". Its occurrence among patients with community-acquired pneumonia has even led to recommendations to include antibiotics active against this organism in the first-line therapy for such patients in some national guidelines for management of pneumonia [22]. In order to improve identification of patients in need of such treatment, and to avoid unnecessary antibiotic therapy, early diagnosis of MP infection is essential.

During the early phase of MP disease, serological methods have low sensitivity and can often only provide a retrospective diagnosis. In contrast, PCR tests on respiratory secretions may provide an early diagnosis for MP infection and could be a useful diagnostic alternative. In this study, we compared PCR from throat swabs and serology in symptomatic patients tested during a community outbreak of MP, and found that PCR on throat swabs detected 93% of patients who later developed an antibody response to MP. In addition, two patients had positive PCR results, but did not develop detectable MP antibodies during follow-up. The superior sensitivity of PCR was noted in patients tested during the first two weeks after onset of illness. Less than half of patients had detectable MP antibodies during this period.

These findings are in agreement with other studies [4,7,10,16-18] and suggest that PCR testing should be considered as the method of choice for diagnosis of MP infection during the early stages of illness.

The drawbacks of PCR are the risks for contamination during processing of the samples or the risk for false negative results due to inhibitory factors in the samples. We purified DNA by the MagnaPure kit, which diminishes the influence of inhibitory factors. The PCR results were reproduced by two independent methods and in addition most patients were positive in two or more samples. Positive PCR results were also corroborated by serological results in all but two cases. These two cases had repeated samples positive by both semi nested PCR and qPCR and seem to represent true MP infections. False positive serological results may also occur, in particular regarding IgM [4]. We had three cases with diagnostic antibody responses but with negative PCR results. In one case the diagnosis was based solely on an elevated IgM titre but the other two cases had significant IgG titre rises in paired sera. The samples for PCR were obtained late for the last two cases, which may explain the discrepant results. The serum sample from the IgM positive cases was obtained during the second week of illness together with a PCR negative throat swab. Whether this represented a false negative PCR or a false positive IgM reaction could not be resolved.

The sensitivity of PCR testing depends on the type of sample tested. We used throat secretions obtained in the oropharynx, since such samples are easier to obtain than nasopharyngeal swabs or sputum samples. Besides, sputum production is often minimal in patients with MP infection. Previous reports have found that sputum samples give the highest rate of positive findings, followed by nasopharyngeal swabs as the second best, with throat swabs appearing to be less efficient. Räty et al. reported a sensitivity of sputum samples of 69%, nasopharyngeal swabs of 50% and throat swabs 37.5% [23]. Nadal et al. observed a sensitivity of 90% for nasopharyngeal swabs as compared to 79% for throat swabs [10]. Throat washings may seem to be better than swabs [24]. However, less distinctive differences have also been reported [25-27].

We used throat swabs in our study, and found that the clinical sensitivity was high, since only 3 of 44 samples from patients with positive MP serology were PCR negative. It is possible that the yield was improved by the fact that our samples were obtained by swabbing the posterior wall of the oropharynx and not the tonsil area.

The interpretation of a positive MP PCR result would be difficult if asymptomatic carriers were common. The highest incidence of MP infection is considered to occur in children 5–15 years of age [28]. To assess the prevalence of asymptomatic carriers in this age group, we performed PCR testing among children in a school at one single day during the outbreak. Only one of the children (0.4%) was found to be positive, and this child was in fact symptomatic. Other studies have shown carrier rates of 0.1–13.5% detected by culture or PCR in healthy subjects during low and high prevalence periods [20,28]. Moreover, no positive cases were found by Dorigo-Zetsma and colleagues [7] among 74 controls or by Kai et al. [8] among 33 healthy volunteers. Our data corroborate previous reports that the frequency of asymptomatic carriers is low in the general population, even during an outbreak of MP infection.

In contrast, among household contacts to patients with confirmed MP infection, we found high rates of MP in throat secretions. All of these subjects had respiratory symptoms. Similarly, Dorigo-Zetsma and co-workers found household members at increased risk of MP infection transmitted by the index case, although not all of these were symptomatic [29].

During longitudinal follow-up, we observed that the majority of PCR-positive patients had persistent infection, even though many of them had received adequate antibiotic treatment. More than half of the patients had detectable MP DNA in throat secretions for seven weeks after onset of illness, and several patients continued to be PCR positive for several months. In some cases, prolonged infection was accompanied by mild respiratory symptoms; however, the significance of MP persistence with respect to such symptoms is difficult to evaluate.

The load of MP DNA gradually declined over time, and eventually all tested subjects became PCR negative. This pattern argues against a transition from active clinical infection into a state of chronic colonization. The persistence of DNA from dead bacteria after antibiotic treatment might occur, but can hardly explain the presence of MP in pharyngeal secretions several months after acute infection. Viable MP bacteria have been isolated by culture for six weeks or longer after resolution of clinical disease [28].

Most of our patients, particularly those who were hospitalized, received antibiotic treatment.

However, in accordance with other investigators [6,19] we could not demonstrate that the rate of clearance was shortened by such treatment. MP adheres to epithelial cells, which seems to be necessary for the pathogenicity of the bacteria. Furthermore, some *Mycoplasma *species such as MP and *M. genitalium *have even been found within host cells. This might suggest that antibiotics with potent intracellular activity are required for resolution of MP infection. *M. genitalium *can be effectively eradicated by azithromycin, but it is not yet known whether that is also the case for MP. Although only an anecdotal observation, in three symptomatic long-term carriers included in our study, MP DNA became undetectable and mild persistent respiratory symptoms disappeared following azithromycin therapy. In addition, other researchers have reported that this drug can reduce transmission of MP [30].

There are several limitations of the present study. The patients were mainly recruited at a hospital department and only few MP cases were detected among patients attending general practitioners. Therefore patients with more severe symptoms may have been overrepresented. The patients are also more likely to have received antibiotic treatment due to the clinical manifestations. The study group mainly includes adults as children are managed at the department of paediatrics.

Our study confirms and reiterates the usefulness of PCR for early diagnosis of MP infection. False negative serological results are common during the first two weeks of illness, but PCR testing of throat swabs from the posterior pharyngeal wall, the oropharynx seem to detect MP at this stage of infection. MP infection is rarely found in healthy people in the community during outbreaks, with the exception of household contacts to confirmed MP cases. MP DNA usually remains detectable by PCR in throat swabs for several weeks after onset of symptoms, and can persist for months, with a gradual decline in bacterial load over time.

## Conclusion

The use of PCR, using either semi-nested or quantitative real-time methods, was superior to serology for diagnosing acute *Mycoplasma Pneumoniae *infections during the first two weeks after onset of illness. During this period, a serological response could be detected only in 23–56% of the cases, whereas PCR detected 96–100% of the cases. During later stages of illness, the rate of positive PCR reactions was lower. A gradual decline in the rate of PCR positive patients was observed over time but half of the patients were still positive 7 weeks after disease onset. All patients eventually became PCR negative but one patient remained positive for seven months. In addition, the bacterial load decreased over time, suggesting gradual clearance rather than a transition from illness to a state of persistent carriage. Only 1/237 school children had a positive MP PCR, and this individual had symptoms of respiratory tract infection at the time of testing. Thus, MP carriage among asymptomatic persons seems to be rare even during an outbreak of MP infection. A positive PCR test for MP should therefore be interpreted as a clinically significant MP infection.

## Authors' contributions

All the authors participated in the design of the study, analysis of the data and the preparation of the manuscript. ACN collected the samples, was responsible for the follow-up of patients and drafted the manuscript. KP performed the laboratory analyses.
